# Maternal serum concentrations of one-carbon metabolism factors modify the association between biomarkers of arsenic methylation efficiency and birth weight

**DOI:** 10.1186/s12940-022-00875-7

**Published:** 2022-07-14

**Authors:** Jeliyah Clark, Paige Bommarito, Miroslav Stýblo, Marisela Rubio-Andrade, Gonzalo G. García-Vargas, Mary V. Gamble, Rebecca C. Fry

**Affiliations:** 1 Department of Environmental Sciences and Engineering, Gillings School of Global Public Health, University of North Carolina, Chapel Hill, North Carolina, USA; 2 Institute for Environmental Health Solutions, Gillings School of Global Public Health, University of North Carolina, Chapel Hill, North Carolina, USA; 3 Department of Nutrition, Gillings School of Global Public Health, University of North Carolina, Chapel Hill, North Carolina, USA; 4 Facultad de Medicina, Universidad Juarez del Estado de Durango, Gómez Palacio, Durango, Mexico; 5 Department of Environmental Health Sciences, Mailman School of Public Health, Columbia University, New York, NY USA; 6 Curriculum in Toxicology and Environmental Medicine, School of Medicine, University of North Carolina, Chapel Hill, North Carolina, USA

**Keywords:** Inorganic arsenic, Birth weight, Gestational age, Effect modification, One-carbon metabolism

## Abstract

**Background:**

Inorganic arsenic (iAs) is a ubiquitous metalloid and drinking water contaminant. Prenatal exposure is associated with birth outcomes across multiple studies. During metabolism, iAs is sequentially methylated to mono- and di-methylated arsenical species (MMAs and DMAs) to facilitate whole body clearance. Inefficient methylation (e.g., higher urinary % MMAs) is associated with increased risk of certain iAs-associated diseases. One-carbon metabolism factors influence iAs methylation, modifying toxicity in adults, and warrant further study during the prenatal period. The objective of this study was to evaluate folate, vitamin B12, and homocysteine as modifiers of the relationship between biomarkers of iAs methylation efficiency and birth outcomes.

**Methods:**

Data from the Biomarkers of Exposure to ARsenic (BEAR) pregnancy cohort (2011–2012)  with maternal urine and cord serum arsenic biomarkers and maternal serum folate, vitamin B12, and homocysteine concentrations were utilized. One-carbon metabolism factors were dichotomized using clinical cutoffs and median splits. Multivariable linear regression models were fit to evaluate associations between each biomarker and birth outcome overall and within levels of one-carbon metabolism factors. Likelihood ratio tests of full and reduced models were used to test the significance of statistical interactions on the additive scale (α = 0.10).

**Results:**

Among urinary biomarkers, % U-MMAs was most strongly associated with birth weight (β = − 23.09, 95% CI: − 44.54, − 1.64). Larger, more negative mean differences in birth weight were observed among infants born to women who were B12 deficient (β = − 28.69, 95% CI: − 53.97, − 3.42) or experiencing hyperhomocysteinemia (β = − 63.29, 95% CI: − 154.77, 28.19). Generally, mean differences in birth weight were attenuated among infants born to mothers with higher serum concentrations of folate and vitamin B12 (or lower serum concentrations of homocysteine). Effect modification by vitamin B12 and homocysteine was significant on the additive scale for some associations. Results for gestational age were less compelling, with an approximate one-week mean difference associated with C-tAs (β = 0.87, 95% CI: 0, 1.74), but not meaningful otherwise.

**Conclusions:**

Tissue distributions of iAs and its metabolites (e.g., % MMAs) may vary according to serum concentrations of folate, vitamin B12 and homocysteine during pregnancy. This represents a potential mechanism through which maternal diet may modify the harms of prenatal exposure to iAs.

**Supplementary Information:**

The online version contains supplementary material available at 10.1186/s12940-022-00875-7.

## Background

Exposure to inorganic arsenic (iAs) is a substantial public health issue. As a result of its natural occurrence in soil and rock, globally, at least 200 million individuals are exposed at harmful levels in contaminated groundwater [1, 2]. In addition to contaminated drinking water, iAs exposure can also occur through diet (e.g., rice intake) [1]. Exposure to iAs is associated with adverse health outcomes [2] and is of particular concern for pregnant women due to associations with lower birth weight and preterm delivery [3–6]. Arsenic crosses the placenta and has been associated with molecular alterations in fetal cells and tissue [7–12]. Potential mechanisms linking prenatal iAs exposure to adverse birth outcomes may include generalized oxidative stress, epigenetic dysregulation of key genes involved in fetal growth, and altered function and expression of genes critical to proper placentation [7–10, 13]. As an example, higher iAs exposure is positively associated with cord blood expression of Soluble Fms-Like Tyrosine Kinase-1 (*sFLT1*), an antagonist of Vascular endothelial growth factor (*VEGF*) [10]. Impacts on the function and expression of genes related to placentation (e.g., *sFLT1*) and fetal growth (e.g., Potassium Voltage-Gated Channel Subfamily Q Member 1 (*KCNQ1*)) [10, 11] observed in relation to iAs exposure could also impair fetal development. Fortunately, modifiable factors influencing iAs metabolism may also be targets for public health intervention.

iAs is metabolized and eliminated from the body through a process that is influenced by micronutrients involved in one-carbon metabolism. This pathway is composed of the folate and methionine cycles and contributes to DNA synthesis, cellular growth, and proliferation [14]. During a critical step in the pathway, the methyl group of 5-methyl-tetrahydrofolate (5-methyl-THF) is transferred to homocysteine by methionine synthase in a reaction that utilizes vitamin B12 as a cofactor. This step generates methionine, an amino acid and component of several compounds [15]. Methionine is then activated to form S-adenosylmethionine (SAM), the primary methyl donor to iAs. Arsenic-3-methyltransferace (AS3MT) sequentially methylates iAs to mono- and dimethyl arsenical species (MMAs and DMAs), using SAM as a cofactor, to drive metabolism and tissue clearance. Inefficient methylation (e.g., higher urinary %MMAs) during pregnancy may indicate higher maternal and fetal exposure to trivalent MMAs^III^, an arsenic species established as the most toxic form in most tissues, including placenta [8, 16, 17].

Each methylation reaction produces a methylated product and S-adenosylhomocysteine (SAH), which is hydrolyzed to homocysteine [15], a non-protein amino acid independently associated with pregnancy complications and intrauterine growth restriction [14]. Given the role of one-carbon metabolism in iAs metabolism, individual susceptibility to iAs toxicity is associated with serum concentrations of folate and vitamin B12 among adults [15, 18, 19]. Impacts during pregnancy, where one-carbon metabolism is already strained to support the demands of the developing fetus, are also likely and warrant further study. To this end, in the present study we explore maternal serum concentrations of one-carbon metabolism factors as potential modifiers of the relationship between biomarkers of methylation efficiency (e.g., % urinary MMAs) and birth outcomes.

We estimate associations between each biomarker and (1) birth weight and (2) gestational age overall and within levels of folate, vitamin B12, and homocysteine using data from the Biomarkers of Exposure to ARsenic (BEAR) pregnancy cohort. Likelihood ratio tests of main effects models against full models incorporating interaction terms were also used to evaluate statistical interactions, or potential effect modification, on the additive scale. We hypothesized that greater bioavailability of folate and vitamin B12 would increase conversion of MMAs to DMAs, thereby attenuating negative associations between biomarkers and birth outcomes.

## Methods

### Study population

The Biomarkers of Exposure to ARsenic (BEAR) cohort comprises pregnant women who resided in Gómez Palacio, State of Durango, Mexico between August 2011 and March 2012 [6]. Arsenic contamination of drinking water is a global public health issue, and the State of Durango is characterized by especially high exposure risk [20]. Mothers were recruited for the study prior to delivery (usually within 24 hours of birth) at the General Hospital of Gómez Palacio, Mexico. Inclusion criteria consisted of: (a) a one-year minimum residence in the Gómez Palacio region, including urban locations of Gómez Palacio and Tlahualilo and their surrounding rural locations, (b) a confirmed, singleton, intrauterine pregnancy without complications (e.g., preeclampsia), and (c) a good overall health status (e.g., no indications of chronic or acute disease). In total, 221 women were approached for the study, and 93% (*n* = 206) provided informed consent for participation. Six of the women providing informed consent were excluded due to a confirmed twin pregnancy (*n* = 1) or sample collection failure (*n* = 5), with the final cohort composed of 200 mother-infant pairs.

Participants completed detailed questionnaires capturing information on demographic characteristics (e.g., time at residence and socioeconomic indicators) and sources of iAs exposure (e.g., sources of drinking, cooking and bathing water). Birth weight was measured at the time of delivery by the physician. Gestational age was estimated using the date of each mother’s last menstrual period. All study procedures were previously approved by the Institutional Review Boards at the University of North Carolina at Chapel Hill and the Universidad Juárez del Estado de Durango, and each participant gave written, informed consent to participate and provide urine samples, drinking water samples, and umbilical cord blood.

### Determination of total iAs and metabolites in urine and cord serum

To characterize maternal exposure to iAs, concentrations of iAs and its metabolites were assessed in spot urine samples. These samples were collected at the time of delivery, immediately transferred to cryovials, and stored in liquid nitrogen. Urine samples packed in dry ice were shipped to the University of North Carolina at Chapel Hill for further processing. To account for differences in water intake or differential hydration, the specific gravity (SG) of each urine sample was measured using a handheld refractometer (Reichert TX 400 #13740000; Reichert Inc., Depew, NY). Additionally, urine iAs (U-iAs) and metabolites (U-MMAs and U-DMAs) were measured using hydride generation atomic absorption spectroscopy (HG-AAS) with cryotrapping [21]. As applied, this method measured total U-iAs, total U-MMAs and total U-DMAs, without differentiating between trivalent (As^III^) and pentavalent (As^V^) species. The limits of detection (LODs) for U-iAs, U-MMAs, and U-DMAs were 0.2, 0.1, and 0.1 μg/L, respectively. These LODs are based on the previously published instrumental LODs [21, 22] and consider volume and dilution of urine samples used for the HG-AAS analysis. Urine samples were adjusted for SG using the following formula: iAs x (mean SG in BEAR cohort-1)/(individual SG-1) [23]. Total urine arsenic (U-tAs) concentrations were estimated as the sum of SG-adjusted U-iAs, U-MMAs, and U-DMAs. Biomarkers of maternal iAs methylation efficiency were calculated as the percent of U-tAs (e.g., U-iAs/U-tAs × 100%).

To characterize fetal exposure to iAs, concentrations of iAs and its metabolites in cord serum (C-iAs, C-MMAs, and C-DMAs) were measured using hydride generation with cryotrapping coupled to inductively-coupled plasma mass spectrometry (HG-CT-ICP-MS), as described previously [24]. This technique provides sensitive LODs, and is thus suited for analysis of biological matrices with very low levels of arsenic species, like serum [25]. LODs for C-iAs, C-MMAs, and C-DMAs were 1.45, 0.06, and 0.12 pg/L, respectively. Additional details on LODs are provided in Laine et al. (2018) [5]. Total cord serum arsenic (C-tAs) concentrations were calculated as the sum of C-iAs, C-MMAs, and C-DMAs.

### Determination of serum concentrations of folate, vitamin B12, and homocysteine

Folate, vitamin B12, and homocysteine were previously measured in maternal serum samples collected between admission to the hospital and the time of delivery [5]. All samples were stored at − 80 °C and shipped to Columbia University, New York, NY for the determination of serum concentrations. Maternal serum folate and vitamin B12 levels were quantified using radio protein-binding assay (SimulTRAC-S; MP Biomedicals, Orangeburg, NY, USA), and homocysteine levels were quantified via high performance liquid chromatography (HPLC) with fluorescence detection [26]. As described previously, and as in other iAs-exposed populations, B12 deficiency was defined at < 128 pM, folate deficiency was defined at < 9 nM, and hyperhomocysteinemia (high homocysteine for non-pregnant populations) was defined at > 10.4 nM [27, 28]. One-carbon metabolism factors were not measured in mothers (*n* = 7) with inefficient amounts of serum (< 200 μL) required for analyses.

### Statistical analysis

Biomarker levels in maternal urine or infant cord serum below the LOD were adjusted using the following formula: LOD/ (√2) [29]. Descriptive statistics were calculated to describe select characteristics of the population. Relationships between variables were estimated using Spearman correlation tests. Correlation tests excluded mothers delivering preterm (*n* = 3) or those missing data on one-carbon metabolism factors (*n* = 7). Unadjusted and adjusted associations between biomarkers of iAs methylation efficiency (%) and first birth weight (grams) and then gestational age (weeks) were estimated overall and within levels of folate, vitamin B12, and homocysteine. Folate, vitamin B12, and homocysteine were dichotomized using clinical cut-offs (e.g., B12-deficient mothers vs. mothers with normal B12) and median splits. Using a directed acyclic graph approach, model covariates were selected for if they were: on an open backdoor path and/or (2) based on their a priori status as determinants of low birth weight [30] (see Additional file 1). The general model form was as follows:

$$birth\ outcome=\alpha +{X}_1\beta +\boldsymbol{Z}\boldsymbol{\beta } +e$$

where *birth outcome* is either birth weight or gestational age. *X*_*1*_ is the biomarker of iAs methylation efficiency (e.g., % MMAs). The *β* coefficient for *X*_*i*_ is the expected mean difference in birth weight or gestational age corresponding to a 1% increase in the respective biomarker. ***Zβ*** is a matrix of potential confounders. Model 1 included the following covariates: maternal age at delivery (years), smoking during pregnancy (0 = no, 1 = yes), and U-tAs (μg/L). Model 2 incorporated the same covariates and folate, vitamin B12, and homocysteine to account for the entire profile of one-carbon metabolism factors. Model 3 incorporated the same covariates as model 1 and excluded women reporting seafood intake within 7 days of providing samples to evaluate potential exposure misclassification resulting from additional sources of organic arsenic species in fish [31]. Models for birth weight excluded mothers delivering preterm (*n* = 3) to reduce confounding by gestational age [32], those missing data on smoking (*n* = 2), and those missing data on one-carbon metabolism factors (*n* = 7), resulting in the final inclusion of 188 mother-infant pairs in unadjusted and adjusted models (174 where C-tAs was evaluated). Models for gestational age excluded mothers missing data on smoking (n = 2) and one-carbon metabolism factors (n = 7), resulting in the final inclusion of 191 mother-infant pairs in unadjusted and adjusted models (177 where C-tAs was evaluated). The leave-one-out approach was also used in a sensitivity analysis to account for the dynamics of arsenic metabolism, as done in other studies [33, 34]. Results from models are presented as the mean difference (95% confidence interval (CI)) in birth weight or gestational age associated with a one-percent increase in each biomarker of iAs methylation efficiency.

Statistical interactions (representing potential effect modification) between biomarkers and one-carbon metabolism factors were also assessed on the additive scale using a likelihood ratio test (LRT) to test whether an interaction model fit the data better than a main effects model, with statistical significance defined at α < 0.10. The (1) interaction and (2) main effect model forms were as follows:

$$(1)\ Birth\ outcome=\alpha +{X}_1\beta +{X}_2\beta +{X}_3\beta +\boldsymbol{Z}\boldsymbol{\beta } +e$$

$$(2)\ Birth\ outcome=\alpha +{X}_1\beta +{X}_2\beta +\boldsymbol{Z}\boldsymbol{\beta } +e$$

where *X*_1_ and *X*_2_ are the biomarker and one-carbon metabolism factor, respectively, and *X*_3_ represents the interaction between the two. *P*-values corresponding to each LRT are displayed alongside estimates from the corresponding stratified model.

## Results

This study includes 200 mother-infant pairs (18–41 years old) participating in the Biomarkers of Exposure to ARsenic (BEAR) pregnancy cohort. Women were recruited in the State of Durango, Mexico, a region characterized by high exposure risk [20]. Select cohort demographics have previously been described [6]. In brief, nearly all (99.0%) women self-reported daily prenatal vitamin supplement intake and 21.7% reported recent seafood intake. Gestational ages ranged from 34 to 42 weeks and three infants were born preterm (< 37 completed weeks of gestation). Fifty-two percent of newborns were male, and the mean birth weight was 3339 g. The prevalence of low birth weight (< 2500 g) was minimal (*n* = 4), and the average birth weight among male and female newborns was 3453 (range of 2100 to 5120) grams and 3215 (range of 1800 to 4200) grams, respectively. The distributions of iAs, MMAs, DMAs, and total arsenic (tAs) measured in urine (U-) and cord serum (C-) are detailed in Table 1. The mean concentration of U-tAs was 37.54 μg/L, ranging from 4.33 to 319.74 μg/L. U-tAs concentrations were also highly correlated with drinking water concentrations of iAs [6]. Biomarkers of iAs exposure were below the limit of detection (LOD) for U-iAs (*n* = 7), U-MMAs (*n* = 4), C-iAs (*n* = 186), C-MMAs (*n* = 127), and C-DMAs (*n* = 54). Since fewer than 30% of samples contained measurements above the LOD for C-iAs and C-MMAs, only C-tAs was used to represent fetal exposure. The mean concentrations of vitamin B12, folate, and homocysteine were 130.97 pM, 40.93 nM, and 6.86 μM, respectively. Two women were deficient in folate (< 9 nM), 142 (74%) women were deficient in B12 (< 128 pM), and 14 (7%) women experienced hyperhomocysteinemia (> 10.4 μM).

**Table 1:** Select demographic and exposure characteristics for the Biomarkers of Exposure to ARsenic (BEAR) cohort (*N* = 200), 2011–2012

Characteristic	n (% non-missing) or mean, median [range]
**Maternal age at delivery (years)**	24, 23 [18–41]
**Smoking status**^a^	
** Nonsmokers**	185 (93.4)
** Current smokers**	13 (6.6)
**Prenatal vitamin daily intake**	197 (99.0)
**Recent seafood intake**^b^	
** No**	155 (78.3)
** Yes**	43 (21.7)
**Gestational age (weeks)**	
** All**	39, 40 [34–42]
** < 37**	3 (1.5)
** ≥ 37**	197 (98.5)
**Newborn sex**	
** Male**	104 (52.0)
** Female**	96 (48.0)
**Birth weight (grams)**	
** All**	3339, 3355 [1800–5120]
** Male**	3453, 3490 [2100–5120]
** Female**	3215, 3150 [1800–4200]
** Low birth weight (< 2500 g)**	4 (2.0)
**Drinking water iAs (μg/L)**	24.6, 13.0 [<LOD^c^–236.0]
**Urinary biomarkers (SG-adjusted)**	
** U-tAs (μg/L)**	37.54, 23.32 [4.33, 319.74]
** U-iAs (%)**	6.08, 5.25 [0.77–45.13]
** U-MMAs (%)**	6.43, 6.02 [0.68–24.86]
** U-DMAs (%)**	87.49, 88.48 [32.68–96.65]
** U-iAs (μg/L)**^d^	2.1, 1.3 [0.14–23.0]
** U-MMAs (μg/L)**^e^	2.3, 1.4 [0.12–18.2]
** U-DMAs (μg/L)**	33.1, 20.6 [1.4–292.5]
**Cord serum biomarkers**	
** C-tAs (pg/mL)**	1.35, 1.24 [1.15–4.0]
** C-iAs (%)**	78.83, 82.86 [25.63–88.96]
** C-MMAs (%)**	4.69, 3.68 [2.90–19.13]
** C-DMAs (%)**	16.48, 13.45 [6.88–67.61]
** C-iAs (pg/L)**^f^	<LOD
** C-MMAs (pg/L)**^g^	<LOD
** C-DMAs (pg/L)**^h^	0.26, 0.17 [<LOD-2.70]
**One-carbon metabolism factors**^i^	
** Folate (nM)**	40.93, 38.59 [7.06–171.46]
** Folate deficient (< 9 nM)**	2 (1.0)
** Homocysteine (μM)**	6.86, 6.37 [4.05–19.42]
** Hyperhomocysteinemia (> 10.4 μM)**	14 (7.3)
** B12 (pM)**	130.97, 117.17 [48.03–747.91]
** B12 deficient (< 128 pM)**	142 (74.6)

^a^Missing data on smoking status for 2 mothers

^b^Missing data on seafood consumption for 2 mothers

^c^limit of detection (LOD) = 0.456 μg/L

^d^*N* = 7 below LOD

^e^*N* = 4 below LOD

^f^*N* = 186 below LOD

^g^*N* = 127 below LOD

^h^*N* = 54 below LOD

^i^*N* = 7 missing data on one-carbon metabolism factors

**Fig. 1:**
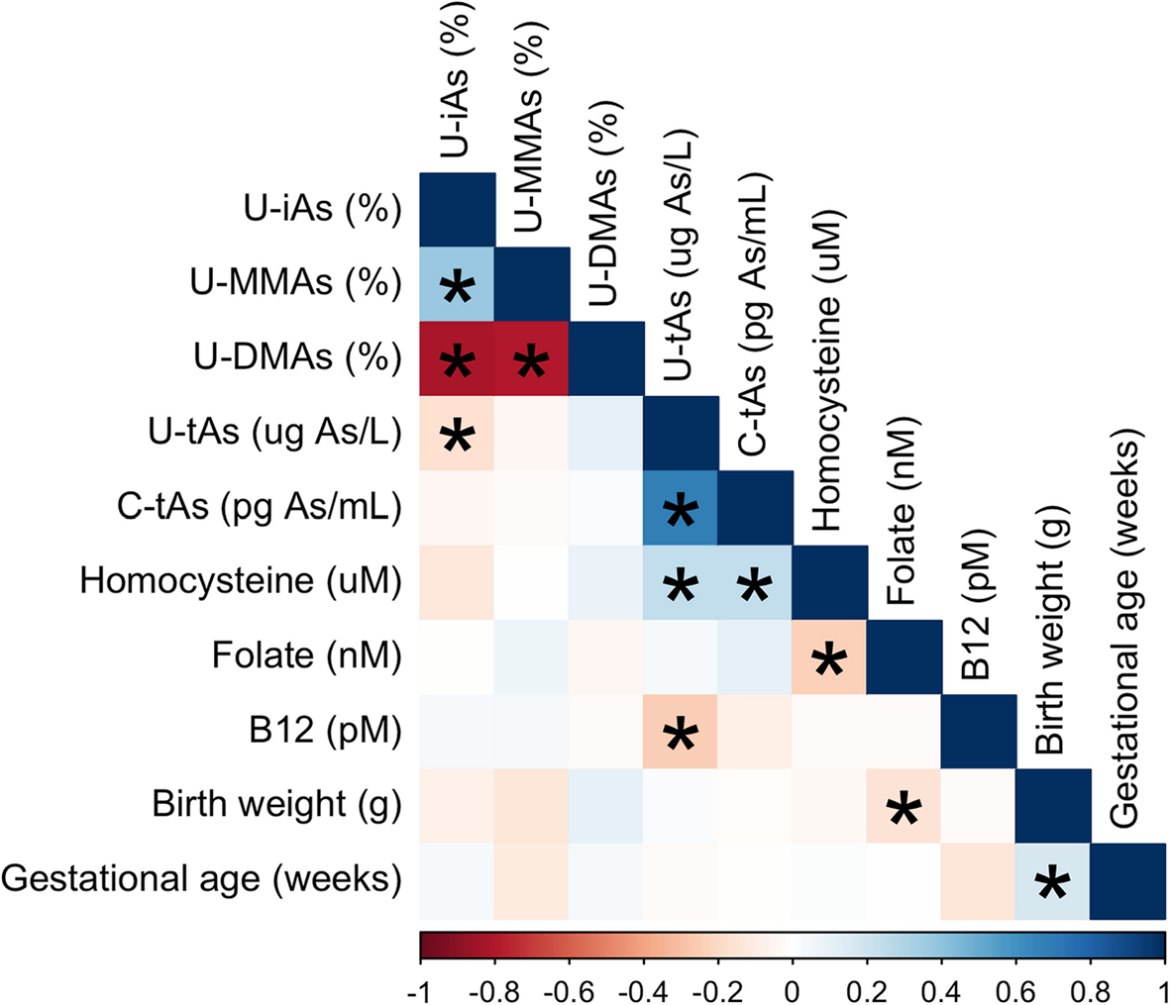
Correlations between biomarkers, one-carbon metabolism factors, and birth outcomes. Excluding preterm births (n = 3) and mothers missing data for one-carbon metabolism factors (*n* = 7). Significant (*p* < 0.05) Spearman correlations are indicated with an asterisk

Generally, biomarkers of iAs methylation efficiency were strongly correlated with each other (Spearman correlation coefficient (ρ) > ±0.60), but the strength of other correlations was weak (*ρ* < ±0.40) (Fig. 1). Vitamin B12 was inversely correlated with U-tAs (*ρ* = − 0.25), while homocysteine was positively correlated (ρ = 0.24). Plasma folate was negatively correlated with homocysteine (ρ = − 0.23) and birth weight (ρ = − 0.15). Birth weight was also negatively correlated with % U-MMAs (ρ = − 0.14). Gestational age was correlated with birth weight (ρ = 0.17) and vitamin B12 (ρ = − 0.13).

**FIG. 2:**
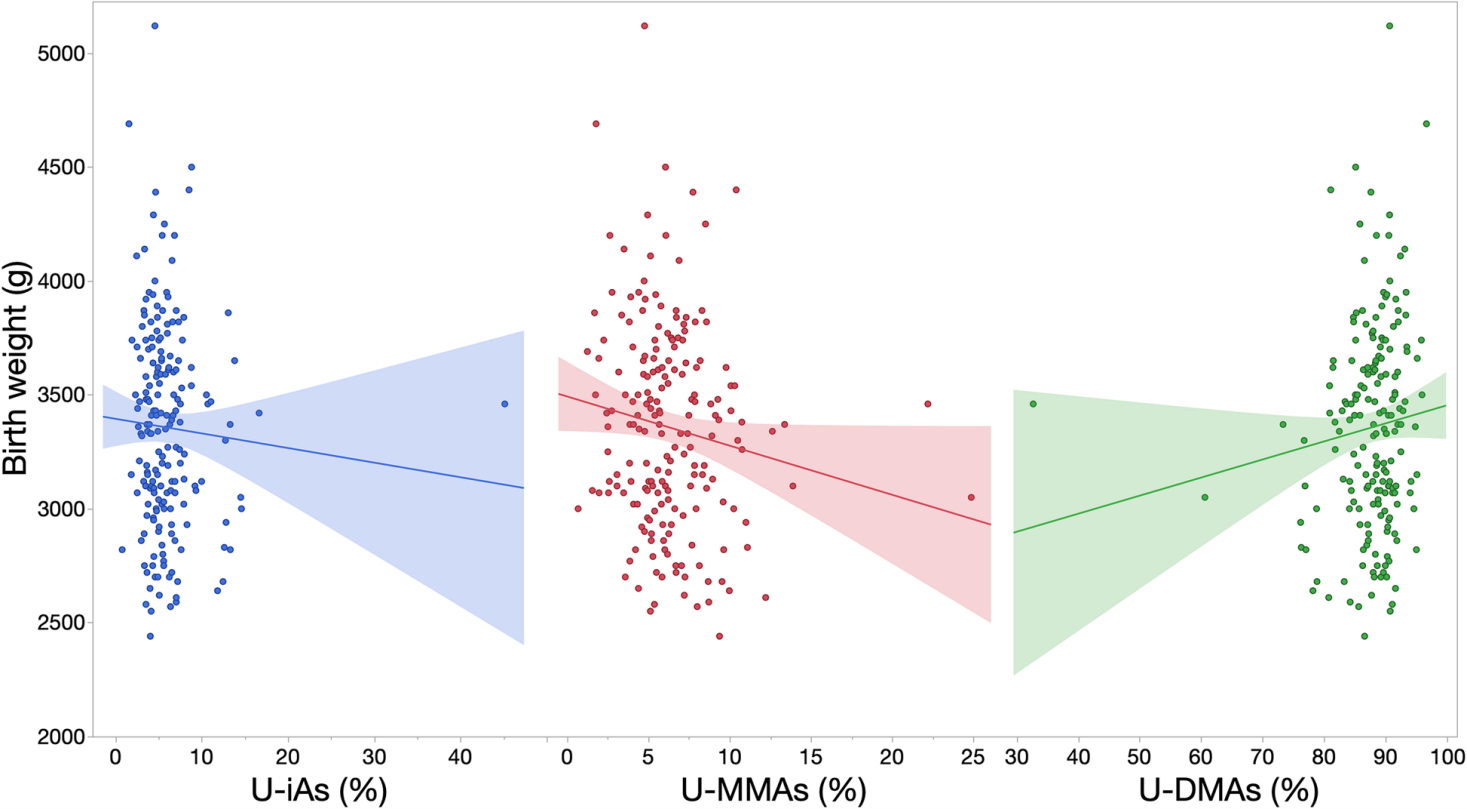
Unadjusted associations (standard error (SE)) between biomarkers and birth weight. Unadjusted linear regression models were fit to model the association (SE) between urinary biomarkers and birth weight, excluding infants born preterm (*n* = 3) and mothers missing data for one-carbon metabolism factors (*n* = 7)

Associations (95% confidence intervals (CI)) between biomarkers of methylation efficiency and birth outcomes were first evaluated across all mother-infant pairs. Among urinary biomarkers, % U-MMAs was most strongly associated with birth weight in unadjusted and adjusted models (β = − 23.09, 95% CI: − 44.54, − 1.64) (Fig. 2**,** Table 2). Associations with C-tAs were greater in magnitude with wider confidence intervals (β = − 233.17, 95% CI: − 577.60, 111.26). Unadjusted associations were generally attenuated by the exclusion of potential outlying values (defined as values above Q3 + 1.5 x IQR or below Q1–1.5 x IQR) in the birth weight and biomarker distributions. Generally, adjustment for potential confounders, one-carbon metabolism factors, or excluding mothers reporting recent seafood intake attenuated associations between biomarkers and birth weight, with the exception of C-tAs, where β estimates became stronger and more negative. Using the leave-one-out approach, we included % U-MMAs as an additional covariate in the % U-DMAs model while adjusting for potential confounders in model 1. With this approach, the β estimate for % U-MMAs was strengthened (β = − 34.37, 95% CI: − 73.65, 4.91) and the β estimate for % U-DMAs shifted from positive to negative (β = − 6.66, 95% CI: − 26.06, 12.74) (see Additional file 2).

**Table 2:** Associations (95% CI) between biomarkers and birth weight (grams) and gestational age (weeks) overall

	Unadjusted β (95% CI)	Model 1^**a**^ β (95% CI)	Model 2^**b**^ β (95% CI)	Model 3^**c**^ β (95% CI)
***Birth weight (grams)***				
** % U-iAs**	−6.11 (− 22.80, 10.58)	−4.61 (− 21.21, 11.98)	−5.55 (− 22.28, 11.19)	− 3.65 (− 21.25, 13.96)
** % U-MMAs**	−20.84 (− 42.65, 0.97)	−23.09 (− 44.54, − 1.64)	− 22.92 (−44.50, − 1.35)	− 21.07 (−45.24, 3.11)
** % U-DMAs**	7.62 (− 3.16, 18.40)	7.56 (− 3.11, 18.22)	7.91 (− 2.82, 18.65)	6.42 (− 5.21, 18.05)
** C-tAs (pg As/mL)**	−51.47 (− 225.84, 122.90)	− 233.17 (− 577.60, 111.26)	−238.72 (− 585.25, 107.82)	− 385.54 (− 892.83, 121.76)
***Gestational age (weeks)***				
** % U-iAs**	0.01 (−0.04, 0.05)	0 (− 0.04, 0.05)	0.01 (− 0.04, 0.05)	0 (− 0.05, 0.05)
** % U-MMAs**	− 0.03 (− 0.09, 0.02)	−0.04 (− 0.10, 0.02)	−0.04 (− 0.10, 0.02)	−0.03 (− 0.10, 0.03)
** % U-DMAs**	0.01 (− 0.02, 0.03)	0.01 (− 0.02, 0.04)	0.01 (− 0.02, 0.04)	0.01 (− 0.02, 0.04)
** C-tAs (pg As/mL)**	−0.06 (− 0.54, 0.41)	0.87 (0, 1.74)	0.86 (− 0.01, 1.74)	0.90 (− 0.31, 2.12)

^a^Model 1 adjusts for smoking status during pregnancy, maternal age, and U-tAs

^b^Model 2 adjusts for smoking status during pregnancy, maternal age, U-tAs, B12, folate, and homocysteine

^c^Model 3 adjusts for smoking status during pregnancy, maternal age, and U-tAs and excludes mothers reporting recent seafood intake

Mean differences in gestational age were less than 0.5-days for urinary biomarkers and in the unadjusted model for C-tAs (Table 2). Neither adjustment for covariates nor exclusion of mothers reporting recent seafood intake had a substantial influence on estimates for urinary biomarkers. In contrast, each one-unit increase in C-tAs was associated with a nearly +1-week mean difference in gestational age after adjustment for covariates (β = 0.87, 95% CI: 0, 1.74) and exclusion of mothers reporting recent seafood intake (β = 0.90, 95% CI: − 0.31, 2.12).

**Fig. 3:**
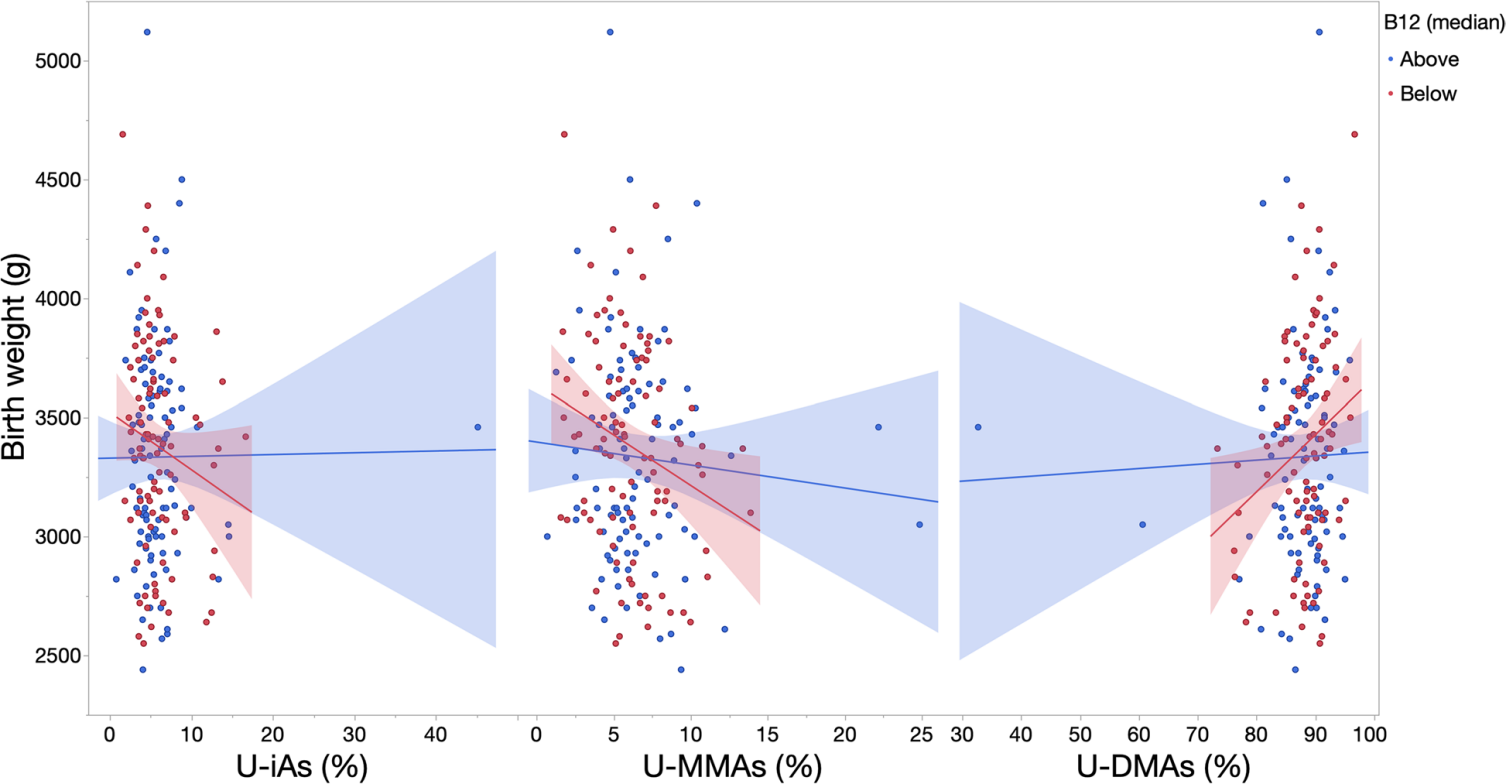
Unadjusted associations (SE) between biomarkers and birth weight within levels of vitamin B12. Unadjusted linear regression models were fit to model the association (SE) between urinary biomarkers and birth weight for each level of vitamin B12 (greater or less than/equal to the median), excluding infants born preterm (n = 3) and mothers missing data for one-carbon metabolism factors (n = 7)

**Fig. 4:**
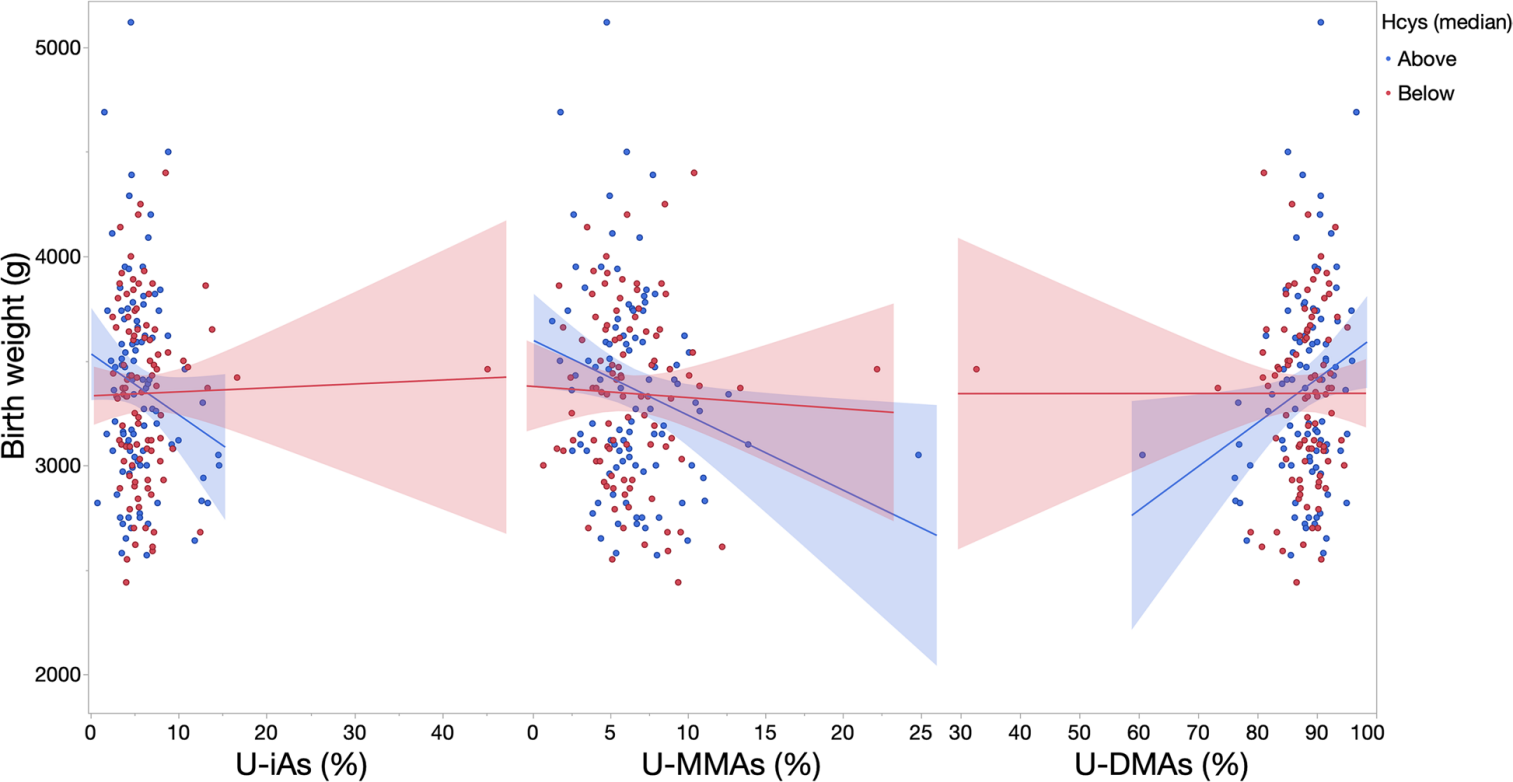
Unadjusted associations (SE) between biomarkers and birth weight within levels of homocysteine. Unadjusted linear regression models were fit to model the association (SE) between urinary biomarkers and birth weight for each level of homocysteine (less or greater than/equal to the median), excluding infants born preterm (n = 3) and mothers missing data for one-carbon metabolism factors (n = 7)

Adjusted models were next stratified by levels of one-carbon metabolism factors to evaluate heterogeneity of effects (Table 3). The negative associations between arsenic variables, including % U-iAs, % U-MMAs and C-tAs, and birth weight were stronger among women with vitamin B12 and folate below the median, as compared to those falling within a healthier range (Figs. 3 and 4). An opposite trend was observed in relation to % U-DMAs, where the negative association between % U-DMAs and birth weight was more positive in relation to lower levels of folate or vitamin B12. As an example shown in Table 3, each one-percent increase in U-MMAs was associated with a − 14.57 g (95% CI: − 57.98, 28.85) mean difference in birth weight among infants born to women with normal B12 levels versus a − 28.69 g (95% CI: − 53.97, − 3.42) mean difference in infants born to B12-deficient mothers.

**Table 3:** Associations (95% CI) between biomarkers and birth weight (grams) within levels of one-carbon metabolism factors

Biomarker	Level	Model 1^a^ β (95% CI)	p	Model 2^b^ β (95% CI)	p	Model 3^c^ β (95% CI)	p
**% U-iAs**	Normal B12	2.07 (−22.72, 26.85)		4.31 (− 20.01, 28.64)		1.08 (−26.06, 28.22)	
	B12 deficient	−16.81 (− 42, 8.37)	0.19	− 18.27 (− 43.57, 7.03)	0.15	− 16.10 (− 43.96, 11.77)	0.30
	Normal homocysteine	−3.12 (− 20.05, 13.81)		− 3.29 (− 20.25, 13.66)		− 2.73 (− 20.69, 15.22)	
	Hyperhomocysteinemia	−79.06 (− 196.3, 38.19)	0.16	− 81.20 (− 215.70, 53.31)	0.15	− 59.58 (− 161.44, 42.27)	0.31
	B12 > median	1.99 (− 18.07, 22.04)		3.61 (− 16.06, 23.28)		0.10 (− 21.56, 21.77)	
	B12 < =median	− 22.79 (− 54.67, 9.09)	0.18	− 24.60 (− 56.30, 7.11)	0.15	−13.66 (− 48.67, 21.35)	0.47
	Folate >median	0.83 (− 17.05, 18.71)		0.81 (− 17.25, 18.86)		−1.27 (− 20.05, 17.51)	
	Folate <=median	−17.57 (− 53.40, 18.26)	0.37	− 17.58 (− 53.55, 18.39)	0.38	− 8.90 (− 49.20, 31.40)	0.71
	Homocysteine <median	**2.07 (− 16.33, 20.46)**		**1.44 (− 16.93, 19.81)**		3.20 (− 16.47, 22.87)	
	Homocysteine > = median	**− 27.96 (− 62.33, 6.41)**	**0.07**	**−31.19 (− 64.67, 2.28)**	**0.07**	− 28.63 (− 67.80, 10.54)	0.12
**% U-MMAs**	Normal B12	− 14.57 (− 57.98, 28.85)		− 6.43 (− 49.81, 36.95)		−17.65 (− 68.03, 32.73)	
	B12 deficient	−28.69 (− 53.97, − 3.42)	0.47	− 28.37 (− 53.63, − 3.10)	0.45	−24.52 (− 53.14, 4.09)	0.71
	Normal homocysteine	−21.71 (− 44.14, 0.72)		− 21.53 (− 44.08, 1.03)		− 21.91 (− 47.24, 3.42)	
	Hyperhomocysteinemia	−63.29 (− 154.77, 28.19)	0.41	−90.66 (− 193.61, 12.30)	0.39	−35.73 (− 119.84, 48.38)	0.76
	B12 > median	− 10.51 (− 38.16, 17.14)		−5.30 (− 32.72, 22.12)		−11.86 (− 43.30, 19.57)	
	B12 < =median	− 46.23 (− 82.26, − 10.19)	0.12	− 42.57 (− 78.65, − 6.49)	0.12	− 41.54 (− 84.48, 1.40)	0.22
	Folate >median	−18.46 (− 43.09, 6.18)		−17.69 (−42.41, 7.03)		−20.58 (− 47.45, 6.29)	
	Folate <=median	−23.45 (− 65.74, 18.84)	0.88	−18.87 (− 62.27, 24.53)	0.94	− 14.34 (− 65.27, 36.59)	0.82
	Homocysteine <median	−8.40 (− 38.64, 21.84)		−9.69 (− 39.89, 20.52)		−11.77 (− 47.49, 23.94)	
	Homocysteine > = median	−33.76 (− 64.90, − 2.61)	0.21	− 28.99 (− 59.88, 1.91)	0.23	−28.20 (−62.70, 6.29)	0.51
**% U-DMAs**	Normal B12	1.25 (−15.84, 18.34)		−1.10 (− 18, 15.80)		1.95 (−17.05, 20.94)	
	B12 deficient	15.72 (0.92, 30.52)	0.12	16.11 (1.30, 30.91)	0.11	14.53 (−2.34, 31.41)	0.23
	Normal homocysteine	6.45 (−4.53, 17.42)		6.45 (−4.55, 17.45)		6.04 (−5.92, 18)	
	Hyperhomocysteinemia	47.81 (−9.56, 105.17)	0.16	57.96 (−5.51, 121.43)	0.15	31.83 (−20.20, 83.86)	0.38
	B12 > median	**1.44 (−11.42, 14.31)**		**−0.37 (−13.05, 12.32)**		2.35 (−11.82, 16.52)	
	B12 < =median	**24.85 (4.27, 45.42)**	**0.05**	**24.22 (3.80, 44.64)**	**0.05**	20.13 (−4.31, 44.57)	0.18
	Folate >median	3.59 (−7.90, 15.08)		3.48 (−8.10, 15.06)		4.70 (−7.52, 16.92)	
	Folate <=median	15.25 (−8.53, 39.04)	0.40	13.78 (−10.32, 37.89)	0.42	8.91 (−19.51, 37.33)	0.76
	Homocysteine <median	**0.50 (−12.34, 13.34)**		**1.04 (−11.79, 13.86)**		0.18 (−13.96, 14.33)	
	Homocysteine > = median	**19.8 (1.47, 38.12)**	**0.05**	**18.86 (0.95, 36.77)**	**0.05**	18.63 (−2.23, 39.49)	0.13
**C-tAs**	Normal B12	−91.24 (− 1287.92, 1105.43)		−20.88 (− 1195.59, 1153.83)		−304.12 (− 1645.15, 1036.91)	
	B12 deficient	− 243.29 (−598.57, 111.98)	0.40	− 230.77 (− 586.09, 124.55)	0.38	− 359.19 (− 932.36, 213.97)	0.41
	Normal homocysteine	− 231.68 (− 582.59, 119.24)		− 239.46 (− 592.17, 113.25)		− 382.58 (− 906.63, 141.46)	
	Hyperhomocysteinemia	−202.33 (− 2782.91, 2378.25)	0.67	−215.25 (− 3567.24, 3136.73)	0.63	− 475.41 (− 2951.40, 2000.58)	0.54
	B12 > median	−254.95 (− 1069.99, 560.09)		− 218.68 (− 1018.69, 581.33)		− 265.88 (− 1216.77, 685.01)	
	B12 < =median	− 204.70 (− 594.89, 185.49)	0.92	−183.57 (− 571.92, 204.78)	0.81	− 425.17 (− 1116.72, 266.38)	0.86
	Folate >median	−238.02 (− 606.24, 130.21)		−210.04 (− 580.34, 160.25)		−313.47 (− 1004.61, 377.68)	
	Folate <=median	−231.13 (− 987.04, 524.78)	0.40	−210.99 (− 970.89, 548.9)	0.42	−475.40 (− 1354.61, 403.81)	0.67
	Homocysteine <median	**− 291.31 (−1004.29, 421.68)**		**−195.95 (− 915.66, 523.77)**		−72.96 (− 967.59, 821.68)	
	Homocysteine > = median	**− 360.07 (− 816.94, 96.80)**	**0.08**	**− 422.54 (− 866.64, 21.56)**	**0.07**	− 597.58 (− 1327.28, 132.12)	0.88

*p*-values represent a likelihood ratio test comparing the main effects model to a full model including an interaction term

^a^Model 1 adjusts for smoking status during pregnancy, maternal age, and U-tAs

^b^Model 2 adjusts for smoking status during pregnancy, maternal age, U-tAs, and additional one-carbon metabolism factors

^c^Model 3 adjusts for smoking status during pregnancy, maternal age, and U-tAs and excludes mothers reporting recent seafood intake

These trends were conserved in relation to homocysteine, where the negative association between biomarkers and birth weight was stronger among infants born to mothers with hyperhomocysteinemia or serum concentrations equal to or greater than the median. As an example, shown in Table 3, each one-percent increase in U-MMAs was associated with a − 21.71 g (95% CI: − 44.14, 0.72) mean difference in birth weight among infants born to women with normal levels homocysteine versus a − 63.29 g (95% CI: − 154.77, 28.19) mean difference among infants born to mothers with hyperhomocysteinemia. Also seen in Table 3, the negative association between biomarkers and birth weight was strongest, yet most imprecise, in relation to C-tAs. Neither adjustment for additional one-carbon metabolism factors nor exclusion of mothers reporting seafood intake had a substantial influence on trends in estimates. Effect modification was significant (likelihood ratio test (LRT) *p*-value < 0.10) on the additive scale for homocysteine (in relation to % U-iAs, % U-DMAs, and C-tAs) and vitamin B12 (in relation to % U-DMAs).

Models evaluating gestational age were also stratified by levels of one-carbon metabolism factors (Table 3). No meaningful associations between urinary arsenic variables and gestational age were observed. However, as seen in Table 3, the association between C-tAs and gestational age ranged from − 0.45-weeks to + 2.87-weeks, albeit with wider confidence intervals. Generally, the positive association between C-tAs and gestational age was stronger among infants born to mothers with higher serum levels of vitamin B12. This trend was conserved in relation to homocysteine. Effect modification was significant on the additive scale for folate (in relation to % U-iAs, % U-MMAs, and % U-DMAs) despite associations not being meaningful Table 4.

**Table 4:** Associations (95% CI) between arsenic biomarkers and gestational age (weeks) within levels of one-carbon metabolism factors

Biomarker	Level	Model 1^a^ β (95% CI)	*p*	Model 2^b^ β (95% CI)	*p*	Model 3^c^ β (95% CI) *N* = 145	*p*
**% U-iAs**	Normal B12	0 (− 0.05, 0.05)		0 (− 0.05, 0.05)		− 0.01 (− 0.06, 0.05)	
	B12 deficient	0.01 (− 0.06, 0.08)	0.87	0.01 (− 0.06, 0.08)	0.93	0.01 (− 0.07, 0.09)	0.97
	Normal homocysteine	0 (− 0.04, 0.05)		0.02 (− 0.03, 0.07)		0 (− 0.05, 0.05)	
	Hyperhomocysteinemia	− 0.02 (− 0.19, 0.15)	0.92	− 0.02 (− 0.10, 0.06)	0.92	− 0.02 (− 0.26, 0.22)	0.90
	B12 > median	0.02 (− 0.04, 0.07)		0.03 (− 0.02, 0.08)		0.01 (− 0.04, 0.07)	
	B12 < =median	− 0.02 (− 0.10, 0.06)	0.36	− 0.06 (− 0.15, 0.04)	0.39	0 (− 0.09, 0.09)	0.65
	Folate >median	**0.02 (− 0.03, 0.07)**		**0 (− 0.04, 0.05)**		0.02 (− 0.03, 0.07)	
	Folate <=median	**− 0.05 (− 0.15, 0.04)**	**0.09**	**− 0.01 (− 0.20, 0.19)**	**0.07**	− 0.05 (− 0.15, 0.06)	0.21
	Homocysteine <median	0.01 (− 0.05, 0.06)		0.01 (− 0.05, 0.06)		0 (− 0.06, 0.05)	
	Homocysteine > = median	− 0.01 (− 0.10, 0.07)	0.63	− 0.01 (− 0.10, 0.08)	0.67	0.01 (− 0.09, 0.11)	0.96
**% U-MMAs**	Normal B12	−0.01 (− 0.11, 0.08)		− 0.02 (− 0.11, 0.08)		− 0.03 (− 0.13, 0.07)	
	B12 deficient	−0.06 (− 0.13, 0.02)	0.35	− 0.06 (− 0.13, 0.01)	0.36	−0.04 (− 0.12, 0.04)	0.72
	Normal homocysteine	−0.04 (− 0.10, 0.02)		0.01 (− 0.07, 0.08)		− 0.04 (− 0.11, 0.03)	
	Hyperhomocysteinemia	0.01 (− 0.13, 0.15)	0.67	−0.09 (− 0.18, 0)	0.57	0.03 (− 0.16, 0.21)	0.66
	B12 > median	0 (−0.07, 0.08)		**0.01 (− 0.06, 0.08)**		0 (−0.08, 0.08)	
	B12 < =median	−0.09 (− 0.17, 0)	0.10	**− 0.11 (− 0.22, 0)**	**0.08**	−0.05 (− 0.16, 0.06)	0.43
	Folate >median	**0 (−0.07, 0.07)**		**−0.04 (− 0.11, 0.02)**		0.01 (− 0.07, 0.08)	
	Folate <=median	**−0.1 (− 0.21, 0)**	**0.08**	**0 (− 0.16, 0.17)**	**0.04**	− 0.12 (− 0.24, 0.01)	0.11
	Homocysteine <median	−0.06 (− 0.15, 0.03)		−0.06 (− 0.15, 0.03)		−0.07 (− 0.17, 0.03)	
	Homocysteine > = median	−0.02 (− 0.10, 0.06)	0.40	−0.02 (− 0.10, 0.06)	0.44	0.01 (− 0.08, 0.10)	0.25
**% U-DMAs**	Normal B12	0 (−0.03, 0.04)		0 (−0.03, 0.04)		0.01 (−0.03, 0.05)	
	B12 deficient	0.02 (−0.03, 0.06)	0.41	0.02 (−0.03, 0.06)	0.44	0.01 (−0.04, 0.06)	0.70
	Normal homocysteine	0.01 (−0.02, 0.04)		−0.01 (− 0.04, 0.02)		0.01 (− 0.02, 0.04)	
	Hyperhomocysteinemia	0 (−0.09, 0.09)	0.90	0.04 (−0.02, 0.09)	0.84	−0.01 (− 0.13, 0.12)	0.89
	B12 > median	−0.01 (− 0.04, 0.03)		− 0.01 (− 0.04, 0.02)		0 (− 0.04, 0.03)	
	B12 < =median	0.04 (− 0.02, 0.09)	0.11	0.06 (0, 0.12)	0.11	0.02 (−0.05, 0.08)	0.42
	Folate >median	**−0.01 (− 0.04, 0.02)**		**0.01 (− 0.02, 0.04)**		**− 0.01 (− 0.04, 0.02)**	
	Folate <=median	**0.06 (0, 0.12)**	**0.03**	**0 (−0.10, 0.11)**	**0.01**	**0.06 (−0.01, 0.13)**	**0.07**
	Homocysteine <median	0.01 (−0.03, 0.05)		0.01 (−0.03, 0.05)		0.01 (−0.03, 0.05)	
	Homocysteine > = median	0.01 (−0.04, 0.06)	0.91	0.01 (−0.04, 0.06)	0.89	−0.01 (− 0.06, 0.05)	0.70
**C-tAs**	Normal B12	1.53 (−0.01, 3.08)		1.50 (−0.04, 3.04)		**1.46 (−0.06, 2.98)**	
	B12 deficient	0.39 (−0.63, 1.41)	0.10	0.38 (−0.65, 1.41)	0.11	**0.18 (−1.47, 1.83)**	**0.06**
	Normal homocysteine	0.94 (0.03, 1.85)		1.91 (0.44, 3.38)		0.99 (−0.28, 2.26)	
	Hyperhomocysteinemia	−0.37 (−3.33, 2.60)	0.77	0.28 (−0.74, 1.31)	0.83	−0.97 (−4.34, 2.40)	0.63
	B12 > median	2.16 (0.70, 3.62)		0.18 (−0.84, 1.20)		**1.90 (0.38, 3.43)**	
	B12 < =median	0.30 (−0.71, 1.31)	0.14	2.41 (0.87, 3.95)	0.11	**0.10 (−1.76, 1.95)**	**0.07**
	Folate >median	0.15 (−0.86, 1.16)		0.96 (0.05, 1.88)		−0.45 (−2.32, 1.41)	
	Folate <=median	2.38 (0.87, 3.88)	0.21	−0.42 (−4.25, 3.41)	0.20	2.34 (0.74, 3.93)	0.86
	Homocysteine <median	2.26 (0.54, 3.98)		**2.32 (0.57, 4.07)**		2.87 (0.91, 4.83)	
	Homocysteine > = median	0.48 (−0.66, 1.62)	0.10	**0.53 (−0.63, 1.68)**	**0.09**	−0.37 (−2.16, 1.42)	0.18

*p*-values represent a likelihood ratio test comparing the main effects model to a full model including an interaction term

*NS* Not significant

^a^Model 1 adjusts for smoking status during pregnancy, maternal age, and U-tAs

^b^Model 2 adjusts for smoking status during pregnancy, maternal age, U-tAs, and additional one-carbon metabolism factors

^c^Model 3 adjusts for smoking status during pregnancy, maternal age, and U-tAs and excludes mothers reporting recent seafood intake

## Discussion

One-carbon metabolism factors modify iAs toxicity and the severity of iAs-associated health outcomes among adults [19, 35], but are understudied during the prenatal period [15]. To this end, we tested effect modification of associations between biomarkers of iAs methylation efficiency and birth outcomes by folate, vitamin B12, and homocysteine in a cohort of mother-infant pairs residing in Durango, Mexico. This region is characterized by high levels of arsenic in drinking water [20], ranging up to 236 μg/L. The average concentration of 37.54 U-tAs (μg/L) measured in the BEAR cohort was an order of magnitude higher than those measured in US pregnancy cohorts (range: 1.96–28.75 μg/L) [36, 37], approaching those observed in highly exposed populations in Bangladesh (range: 5.1–325 μg/L) [38, 39]. Effect modification by vitamin B12 and homocysteine was significant on the additive scale for some associations with birth weight. Using stratified models, we found that the negative association between arsenic biomarkers and birth weight was stronger among infants born to mothers with lower levels of vitamin B12 or folate. Findings for gestational age were less compelling, as meaningful differences were only observed with respect to C-tAs. These findings highlight a potential role for maternal diet in modifying iAs-associated lower birth weight.

Seventy-four percent of mothers in the BEAR cohort were deficient in vitamin B12, a clinical condition independently associated with maternal and neonatal outcomes, including impaired fetal growth [40]. The relationship between vitamin B12 intake and the distribution of iAs and metabolites has varied between studies (as reviewed in [15, 41]), but we previously identified higher mean concentrations of U-tAs in B12-deficient mothers in the BEAR cohort [5]. Higher levels of MMAs in maternal urine were negatively associated with birth weight and placenta weight in this cohort [6]. Since MMAs^III^ is established as the most toxic arsenic species in most tissues including placenta [8, 16, 17], it is notable that we identified stronger negative associations between % U-MMAs and birth weight among infants born to women with lower B12 levels. Among infants born to mothers with B12 levels below the median, a 10% increase in % U-MMAs was associated with a mean difference in birth weight exceeding that associated with smoking 6 to 10 cigarettes daily during pregnancy [42]. Group differences were generally more pronounced between strata of vitamin B12, as compared to between strata of folate. According to the “methylfolate trap” hypothesis, B12 deficiency prevents a) the remethylation of homocysteine to methionine, thereby reducing SAM synthesis, and b) the metabolism of 5-methyl-THF to THF. This causes 5-methyl-THF, homocysteine and SAH to accumulate in cells and presents as a functional folate deficiency [40]. Given the high prevalence of mothers with B12 deficiency, this functional folate deficiency may explain why group differences were more pronounced between strata of vitamin B12. Also, overt B12 deficiency was highly prevalent, whereas few participants appeared to be overtly folate deficient, even in the low folate strata. Nonetheless, higher levels of folate were protective against iAs-associated lower birth weight in this study, as seen in prior studies conducted in pregnant populations using single [43] and multi-nutrient [44] approaches.

The present study is among the first to examine homocysteine in relation to iAs-associated lower birth weight. We observed stronger and more negative associations between % U-iAs, % U-MMAs and C-tAs and birth weight in higher maternal homocysteine strata. Homocysteine is a metabolite of one-carbon metabolism that is independently associated with preeclampsia, intrauterine growth retardation, and other adverse pregnancy outcomes [45, 46]. Prior research in adult populations exposed to iAs highlight a relationship between higher levels of homocysteine and lower % DMAs [47, 48], reflecting incomplete methylation of iAs to DMAs. It is also speculated that homocysteine competitively inhibits transport of other amino acids to impact syncytiotrophoblast metabolism and function, as well as the supply of nutrients to the fetus [14].

Associations between C-tAs and birth outcomes were larger and more imprecise, as compared to urinary biomarkers. Generally, the association between C-tAs and gestational age was more positive among infants born to mothers with higher vitamin B12 or lower homocysteine levels, but less consistent for associations with birth weight. Larger, more imprecise estimates likely reflect the narrow range of C-tAs (1.15 to 4.0 pg As/mL). Inference is further complicated by the use of left-censored data, given the number of samples below the LOD for cord serum arsenicals.

This study is not without limitations. Given its size, the BEAR cohort is underpowered to detect statistical interactions. Moreover, stratified analyses required even smaller sample sizes, decreasing precision. Second, one-carbon metabolism factors were assessed at the end of pregnancy and maternal plasma levels are altered over the course of pregnancy in order to support fetal growth [14]. Early pregnancy may be a more critical window of susceptibility given the association between iAs exposure and the expression of genes related to placentation and imprinting [10, 11]. Given the cross-sectional design of the study, there is also a potential for reverse causation, where iAs may hinder one-carbon metabolism. Additionally, protein intake and maternal body mass index (BMI) are important potential confounders not measured in this study. We previously identified a positive association between BMI and % U-DMAs among adults [49], and have also identified a relationship between pre-pregnancy BMI and the expression of genes underlying nutrient metabolism in the placenta [50]. Despite 74% of mothers in the BEAR cohort being deficient in vitamin B12, nearly all reported daily prenatal vitamin intake. This disparity may reflect recall bias or the consistent decrease in serum vitamin B12 that occurs throughout pregnancy as it is transported across the placenta to the developing fetus [51–53]. Future studies should utilize longitudinal data with repeated urinary measures and capture potential sources of arsenic exposure (e.g., drinking water and/or diet). Finally, we evaluate biomarkers of prenatal iAs methylation individually, but mixtures can exert joint effects that differ from individual species. Multi-nutrient deficiencies are also prevalent in undernourished populations [54], limiting the generalizability of our findings to other iAs-exposed populations, especially those in regions characterized by food insecurity.

## Conclusions

In a cross-sectional study, we found that associations between % U-MMAs and birth weight were attenuated by elevated levels of vitamin B12 and folate in maternal serum. These trends were also observed using strata defined by clinical cut-offs. Differences in rates of conversion of MMAs to DMAs may occur according to levels of one-carbon metabolism factors. This study may have public health implications as it positions B vitamins as potential modifiers of the harms of exposure to iAs during pregnancy, but validation is needed given the limitations of our study design. In most arsenic-endemic regions, folic acid fortification is not mandated [19]. Vitamin B12 deficiency is also prevalent in regions characterized by iAs-contamination of drinking water [40]. Future studies should measure arsenic biomarkers, micronutrients, and fetal growth parameters in early pregnancy and across each trimester to capture the dynamic relationship between one-carbon metabolism, fetal development, and iAs exposure that may not be observed in this study. The incorporation of unmeasured potential confounders (e.g., additional micronutrients, energy and protein intake, and BMI) and social factors will also be important given the reality of interactions between environmental and structural factors. Though we demonstrate potential protection against the harms of prenatal arsenic exposure by B vitamins, efforts supporting remediation in drinking water should remain priority.

## Supplementary Information

**Additional file 1: Figure S1.** Simplified directed acyclic graph (DAG).

**Additional file 2: Table S1.** Results from models fit using the leave-one-out approach.

## Data Availability

The datasets used or analyzed during the current study are available from the corresponding author on reasonable request.

## Abbreviations

iAs: Inorganic arsenic
MMAs: Monomethylated arsenic
DMAs: Dimethylated arsenic
LRT: Likelihood ratio test
SAM: S-adenosylmethionine
5-methyl-THF: 5-methyl-tetrahydrofolate
SAH: S-adenosylhomocysteine
BEAR: Biomarkers of Exposure to ARsenic
SG: Specific gravity
U-tAs: Total urinary arsenic
U-iAs: Urinary inorganic arsenic
U-MMAs: Urinary monomethylated arsenic
U-DMAs: Urinary dimethylated arsenic
C-tAs: Total cord serum arsenic
C-iAs: Cord serum inorganic arsenic
C-MMAs: Cord serum monomethylated arsenic
C-DMAs: Cord serum dimethylated arsenic
HG-CT-ICP-MS: Hydride generation with cryotrapping coupled to inductively coupled plasma mass spectrometry
LOD: Limit of detection
CI: Confidence interval
*sFLT1*: Soluble Fms-Like Tyrosine Kinase-1
*VEGF*: Vascular endothelial growth factor
*KCNQ1*: Potassium Voltage-Gated Channel Subfamily Q Member 1
DAG: Directed acyclic graph
