# Supplementary figures and images for: Maternal serum concentrations of one-carbon metabolism factors modify the association between biomarkers of arsenic methylation efficiency and birth weight
Source: Environ Health. 2022 Jul 14;21:68. doi: 10.1186/s12940-022-00875-7 (PMC9281096; doi:10.1186/s12940-022-00875-7)

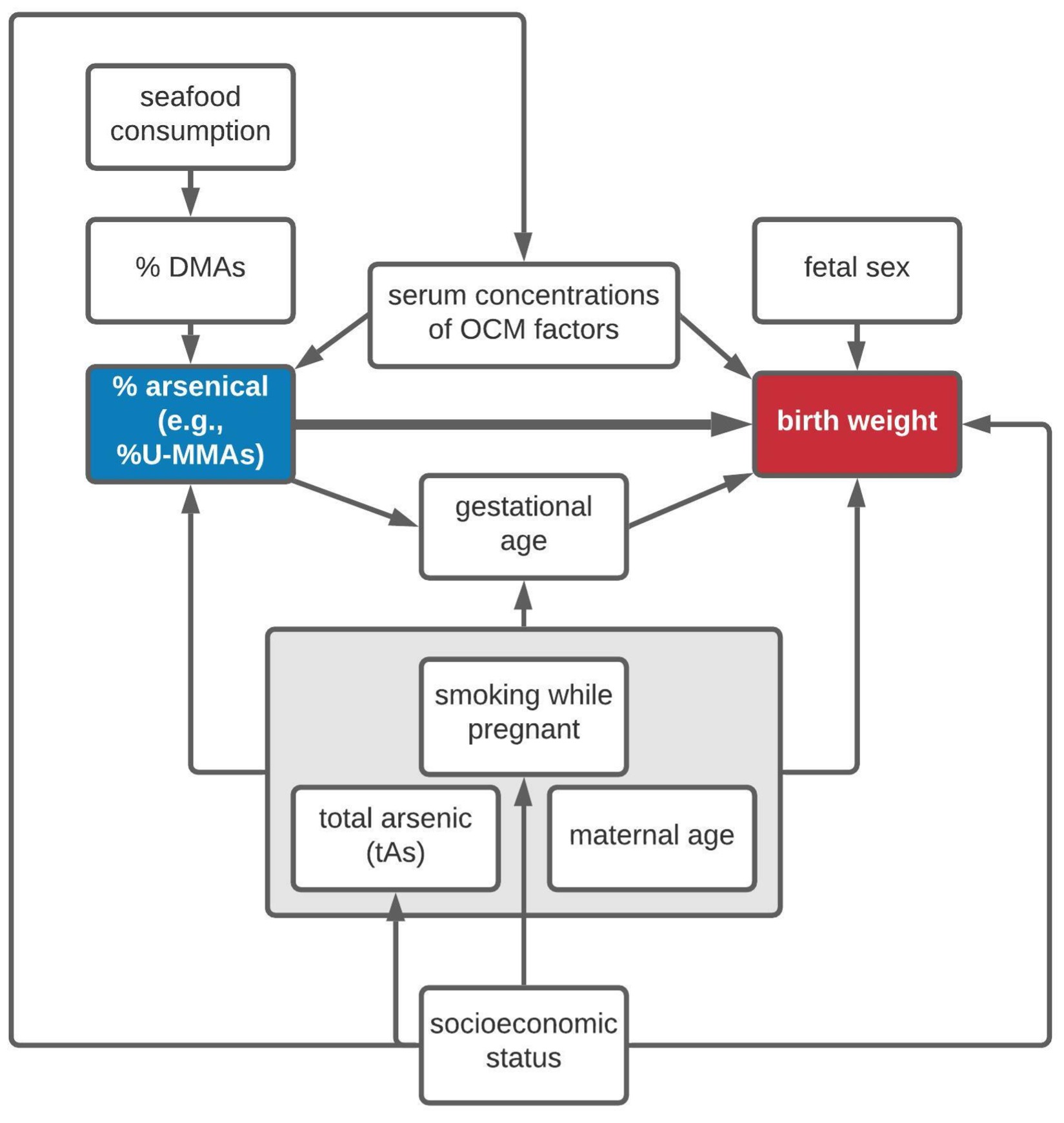

Supplement: Supplementary file 1 — Additional file 1: Figure S1. Simplified directed acyclic graph (DAG). [file 12940_2022_875_MOESM1_ESM.jpg]
